# Improving online and offline gain from repetitive practice using anodal tDCS at dorsal premotor cortex

**DOI:** 10.1038/s41539-021-00109-4

**Published:** 2021-10-22

**Authors:** Taewon Kim, John J. Buchanan, Jessica A. Bernard, David L. Wright

**Affiliations:** 1 Department of Neurology, Duke University School of Medicine, Durham, NC 27710 USA; 2 Department of Kinesiology, Texas A&M University, College Station, TX 77845 USA; 3 Texas A&M Institute for Neuroscience, Texas A&M University, College Station, TX 77845 USA; 4 Department of Psychological and Brain Sciences, Texas A&M University, College Station, TX 77845 USA

**Keywords:** Premotor cortex, Consolidation

## Abstract

Administering anodal transcranial direct current stimulation at the left dorsal premotor cortex (PMd) but not right PMd throughout the repetitive practice of three novel motor sequences resulted in improved offline performance usually only observed after interleaved practice. This gain only emerged following overnight sleep. These data are consistent with the proposed proprietary role of left PMd for motor sequence learning and the more recent claim that PMd is central to sleep-related consolidation of novel skill memory.

## Introduction

Transcranial direct current stimulation (tDCS) can modulate cortical excitability and facilitate skill acquisition. To date, most studies administering tDCS during the practice of motor skills have focused on a role for the primary motor cortex (M1). Far fewer have targeted other motor planning sites critical for skill acquisition^1^. Recently, anodal tDCS was used to examine a causal link between M1^2^ and SMA^3^ with the extent of offline skill enhancement for multiple novel motor skills following interleaved practice (IP) and repetitive practice (RP) formats. The choice of neural targets for exogenous stimulation in these studies was based on a report from functional imaging work of heightened activity at M1, SMA, and PMd during IP being associated with greater offline gain in skill^4^.

**Fig. 1:**
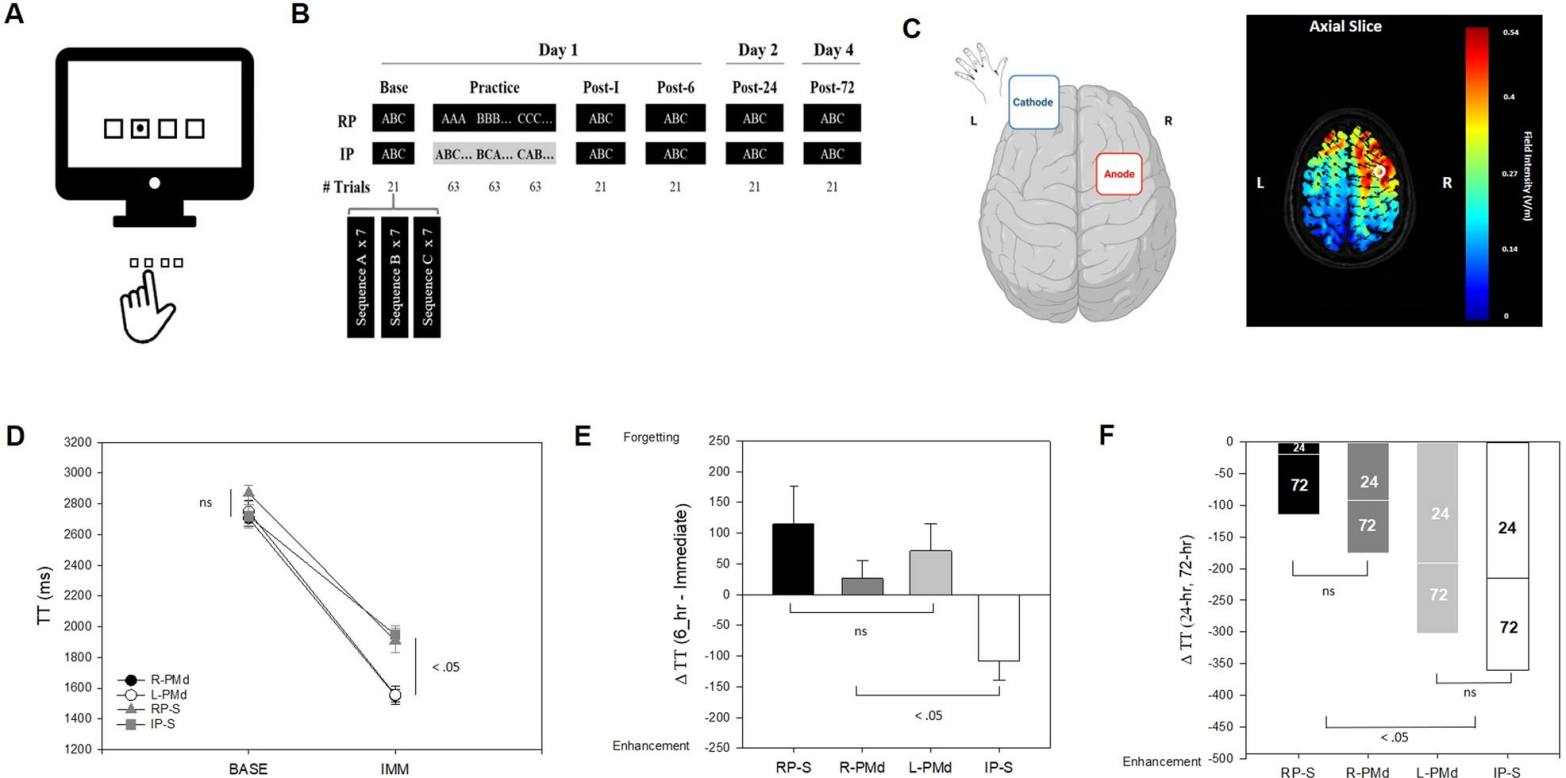
The experimental procedure with tDCS montage used to examine motor memory enhancement. Participants in the present study practiced three unique six-element motor sequences that were executed using the left index finger only with each key-press in the sequence made in response to a visual signal presented on the display (**A**). In the present work, repetitive practice (RP) involved the practice of a single sequence for a total of 63 trials prior to practice with another sequence. There were three sequences practiced in the RP condition. Interleaved practice (IP) involved 21 trials for each of the three to-be-learned motor sequences in each block of 63 trials. All test blocks (Base, Post-I, Post-6, Post-24, Post-72) involved seven trial for each sequence practice in a RP format (**B**). Anodal tDCS at right or left PMd was administered for individuals experiencing RP and consisted of the anode being located 2.5 cm anterior of M1 with a reference electrode at the supraorbital region (figure illustrates right PMd montage only). The human brain icon was adapted by Biorender.com. The current flow associated with this electrode montage was modeled using tDCS-Explore^TM^ (Soterix Medical Inc., New York, NY) (figure illustrates right PMd only). Heightened current flow was observed (.229 V/m, .406 V/m) at MNI coordinates (x: −29, y: −1, z; 44 and x: 29, y: −1, z; 44). These locations (noted with white circle) fell within the boundaries described as left-PMd and right-PMd respectively in the human motor area template^12^ (**C**). Online gain, reflected in the change in TT from the test administered prior to practice and one given immediately after the conclusion of training, did not differ as a function of the location of anodal tDCS at PMd but was significantly greater than that observed for individuals in RP-S and IP-S (**D**). The change in TT during the initial 6-hrs following RP supplemented with stimulation at PMd did not differ from that observed for the individuals that experienced RP-S but differed from the performance of individuals exposed to IP-S who revealed enhanced skill memory across the 6-hr interval after practice (**E**). While anodal tDCS at R-PMd failed to induce sleep-related performance improvement beyond that observed for RP-S, stimulation at L-PMd resulted in overnight skill memory enhancement congruent with that observed for the IP-S condition. This occurs following the initial night of sleep as well as for the subsequent assessment made following two additional nights of sleep **(F)**. RP-S Repetitive practice paired with sham stimulation, IP-S Interleaved practice paired with sham stimulation, L-PMd Repetitive practice paired with anodal tDCS at left PMd, R-PMd Repetitive practice paired with anodal tDCS at right PMd. tDCS transcranial direct current stimulation. All error bars are standard errors.

These initial investigations focused on upregulating activity at contralateral M1 or SMA during RP, using anodal tDCS, in an attempt to induce offline gain more typical of IP. Specifically, anodal tDCS was administered at M1 or SMA during 20-min session of RP as novel motor sequences were performed with the left index finger (Fig. 1A, B). Retention tests, in the absence of any stimulation, were administered 6-h post practice as well 24-h later after an overnight sleep. Stimulation at both M1 and SMA during RP, led to significant offline gain compared to the sham counterpart. However, the manner in which this offline behavioral gain was manifest was dependent on the location of stimulation during RP. Anodal tDCS at M1 resulted in broad gains in skill memory consistent with those observed after IP^2^. That is, rather than the usual significant forgetting immediately after RP, early consolidation emerged reflected in the stable performance exhibited across a 6-h post practice test interval. This was accompanied by a subsequent enhancement in skill memory following overnight sleep in the absence of further RP. These data are congruent with the proposed role of M1 during early consolidation^5^ as well as for sleep-related offline gain^6^ in cases where only a single motor skill is acquired. In contrast, when tDCS was applied at SMA^3^, the offline benefit that emerged was limited to improved performance after overnight sleep, a finding consistent with the claim that SMA is central to sleep-dependent consolidation of motor sequences^7^.

The present investigation extended these earlier efforts by examining the role of PMd for the consolidation of multiple novel motor memories acquired via RP. This work was based on recent reports that recruitment of PMd is central to the retention advantage typically associated with exposure to a training in an IP format^8^.

**Main Text**

Separate experimental conditions, each involving 16 participants (Table 1) involved the administration of anodal tDCS at either left (L-PMd) or right PMd (R-PMd) throughout RP and as sham (RP-S, IP-S). Participants were right-handed undergraduate (between 19 and 23 years old) students (*N* = 64, Males: 29, Females: 35) that received course credit for their participation. Individuals had no prior experience with the experimental tasks and were unaware of the specific purpose of the study. All individuals that participated in this study had no history of epilepsy, any known neurological disorder, no psychiatric history, were medication-free during the previous 14-days prior to participation, had not used alcohol within the previous 24-h and were not pregnant. All participants completed an informed written consent approved by Texas A&M University’s Institutional Review Board before any involvement in the experiment.

**Table 1:** Gender and participant distribution to each of the four experimental conditions used in this experiment.

	AtDCS at left PMd		AtDCS at right PMd		Sham RP		Sham IP	
Gender	Male	Female	Male	Female	Male	Female	Male	Female
# of participants	7	9	9	7	7	9	6	10

**Fig. 2:**
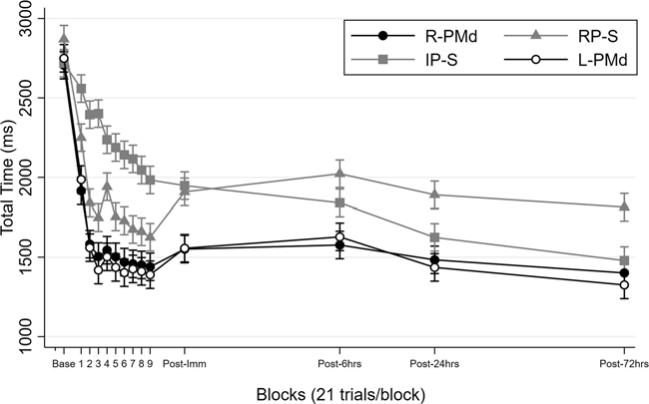
Total time (ms) for the nine training blocks performed between the baseline (BASE) and immediate posttest (Post-Imm) blocks during which real (R-PMd, L-PMd) or sham (RP-S, IP-S) stimulation was administered during repetitive training (R-PMd, L-PMd, PR-S) and interleaved training (IP-S). No stimulation was present during any tests including 6-h post (Post-6 h), 24-h post (Post-24 h), and 72-h post (Post-72 h). Error bars are standard errors.

Participants in all conditions performed similarly during the test administered prior to any practice and stimulation, *F*(3,60) = 1.32, *p* = 0.28, η_*p*_^2^ = 0.06 (Fig. 1D). Immediately after practice, TT was similar for individuals exposed to anodal tDCS at either right or left PMd during RP which in turn was significantly lower than TT for individuals in the sham conditions, *F*(3,60) = 11.01, *p* < 0.001, η_p_^2^ = 0.36. Subsequent post-hoc assessment of the performance during the IMM test revealed that individuals that received anodal stimulation at left PMd (*M* = 1556 ms, *SEM* = 60 ms) and right PMd (*M* = 1551 ms, *SEM* = 42 ms) did not differ (*p* = 0.96), but mean TT for both stimulation conditions were significantly lower than mean TT for the individuals assigned to the RP-Sham (*M* = 1909 ms, *SEM* = 78 ms) and IP-Sham (*M* = 1949 ms, *SEM* = 59 ms) conditions (*p* < 0.001). Performance for the sham conditions during the IMM test did not differ significantly (*p* = 0.67) (Fig. 1D). The 4 (Condition: RP-Sham, IP-Sham, R-PMd, L-PMd) x 9 (Block: 1–9) mixed-model ANOVA with repeated measures on the last factor on mean TT from the training blocks during which real and sham stimulation was present revealed significant main effects of Condition, *F*(3,60) = 41.07, *p* < 0.001, η_p_^2^ = 0.67, and Block, *F*(8, 480) = 101.08, *p* < 0.001, η_p_^2^ = 0.39. This analysis also revealed a significant Condition × Block interaction, *F*(24, 480) = 3.67, *p* < 0.001, η_p_^2^ = 0.16. Simple main effect analysis of this interaction revealed a couple of noteworthy findings, (a) real stimulation at right and left PMd led to a similar performance that was in turn superior for almost all training blocks (exception Block 9) compared to the sham conditions, (b) the RP-sham condition exhibited a significantly quicker reduction in mean TT compared to the IP-Sham condition (Fig. 2). Most critically, these data suggest that upregulation of PMd via anodal tDCS in RP during training impacts the development of novel motor memories.

Stimulation at PMd during RP however failed to induce time-dependent consolidation that is typical of IP but absent after RP during the initial 6-hr following practice, *F*(3,60) = 4.82, *p* < 0.01, η_p_^2^ = 0.19. Specifically, post hoc assessment revealed that the change in TT from the immediate to 6-h test was similar for R-PMd, L-PMd, and the RP-S conditions but differed from the IP-S condition which was the only condition that exhibited an improvement in performance after only 6-hrs post-practice (Fig. 1E). One additional finding that is particularly noteworthy is the rather dramatic change in performance (i.e., increase in TT) of the participants in the RP-S condition at the time of the post-immediate test (Fig. 2). On the surface this was unanticipated given the trials during both the training blocks and the tests are basically identical. It should be noted however that this test occurred after approximately 3–5 min which was the approximate time to remove the tDCS electrodes, re-situate the participant to complete these trials, as well as complete a post-training questionnaire regarding adverse effects. The fact that performance deteriorates in this manner suggests that the forgetting commonly observed following RP formats is incredibly rapid, further supporting the common claim that exposure to this practice scheduling condition is particularly ineffective for retention. This finding has probably gone unnoted in many previous studies because tests are most commonly administered after at least 24-h. More importantly, this finding appears robust as we observed a similar increase in TT across this interval in two other recent papers that included the RP-S condition^2,3^.

As expected, IP-S resulted in significantly greater offline gain following overnight sleep compared to that observed for individuals in the RP-S condition. While the receipt of anodal tDCS at R-PMd during RP did little to improve consolidation that occurred overnight beyond that displayed by RP-sham, the same stimulation at L-PMd facilitated performance following sleep in a manner similar to that observed for IP-S, *F*(3,60) = 2.85, *p* < .05, η_p_^2^ = 0.12 (Fig. 1F). These data revealed that the individuals in the L-PMd and IP-S indicated significantly greater sleep-related offline gain compared to their R-PMd and RP-S counterparts (*p* < 0.05). These data then highlight different roles for left and right PMd when activation of these neural regions is upregulated, in this case via exogenous stimulation, during RP.

As a whole, these data provide novel evidence that neuromodulation of targets beyond M1, via application of anodal tDCS during practice, can facilitate not just the acquisition and retention of a single novel motor skill but also multiple skill memories developed simultaneously. Stimulation targeting left PMd during RP of three novel motor sequences resulted in an overnight offline performance gain only previously observed following IP^4^. These data are consistent with the recent report of a role for neural circuits that include PMd during sleep-related consolidation^9^. Moreover, the absence of this overnight gain from stimulation targeting right PMd is congruent with the claim for a proprietary role for left PMd for motor sequence learning^10,11^. Finally, the present work revealed superior acquisition when RP was supplemented with anodal tDCS targeting PMd suggesting that neural circuitry that include this region can influence, not only the maintenance of novel skill memory (i.e., in the case of L-PMd) but contribute to the initial formation of these memories.

## Methods

### Transcranial direct current stimulation (tDCS)

Real stimulation consisted of a 2 mA current applied via a 25 cm^2^ (5 × 5 cm) anode and 35 cm^2^ (5 × 7 cm) reference electrode covered by saline-soaked sponges resulting in a maximum current density of 0.08 mA/cm^2^ administered using a 9 V battery-driven stimulator (tDCS Stimulator; TCT Research Limited, Hong Kong). The anode was located 2.5 cm anterior to M1 (i.e., FC3 or FC4, International 10–20 system) and was paired with a reference electrode at either right or left supraorbital site. The current flow associated with this electrode montage was modeled using tDCS-Explore^TM^ (Soterix Medical Inc., New York, NY) and revealed heightened current flow at regions described as right and left PMd in the human motor area template (Fig. 1C)^12^. In addition to the participants assigned to RP with stimulation at right or left PMd, two additional sham conditions, each including a separate group of 16 participants, received training in either a RP (RP-S) or IP (IP-S) format. Real (L-PMd, R-PMd) or sham (RP-S, IP-S) stimulation was applied during the entire 20-min period of practice. The current began in a ramp-like fashion over 30-s until reaching 2 mA. For the real stimulation conditions, the current was maintained for the entire 20-min practice period. In contrast, for the Sham conditions the current was ramped down after 30-s. Participants were blinded to the stimulation condition but no formal analyses were conducted to evaluate the blinding process^13^. At the conclusion of the experiment adverse effects from tDCS application were assessed via questionnaire. No adverse responses were reported by participants from all experimental conditions.

### Experimental procedure

All individuals completed 63 trials for each of three six-key motor sequences executed with the left index finger on a standard keyboard resulting in 189 trials of practice in either a repetitive or interleaved practice schedule (Fig. 1A). A participant experienced nine blocks of 21 trials with each block in RP containing trials with only a single motor sequence. In contrast, for IP, each block consisted of seven trials of each of the three motor sequences that were being acquired. Test blocks were conducted in the absence of any stimulation in a RP format for all participants and were administered prior to (BASE) and immediately (IMM) after training, as well as 6-h, 24-h, and 72-h later (Fig. 1B). Individuals executed a key-press to a visual signal that was spatially compatible with the position of the key. Once a correct key was pressed, the next visual signal in the sequence was presented^14^. The primary dependent variable assessing motor sequence performance was the total time (TT) which was the interval from the presentation of the first stimulus to the correct execution of the final key-press of the motor sequence. Since mean TT was not normally distributed, for all of the analyses present below, the median TT was used as an estimate of performance for each individual for each training and test block.

### Statistical analyses

Evaluation of performance during training (i.e., online performance) was assessed in two separate ways. First, the impact of being exposed to stimulation during repetitive practice (RP) during the tests performed prior to (BASE) and immediately after training (IMM) was completed was examined using a 4 (Condition: RP-S, IP-S, L-PMd, R-PMd) × 2 (Test: BASE, IMM) mixed-model analysis of analyses (ANOVA) with repeated measures on the last factor. Second, online performance during the trials during which real or sham stimulation was experienced by the participants using a 4 (Condition: RP-S, IP-S, L-PMd, R-PMd) × 9 (Block: 1–9) mixed-model ANOVA with repeated measures on the last factor. Significant interactions were subsequently assessed using simple main effect tests and Student–Newman–Keuls post hoc assessment was used where necessary.

To evaluate offline gains (loss), difference scores (DS) for each individual were determined for (a) a time-dependent gain (loss) was calculated based on the difference in TT between the IMM and 6-h tests, and (b) sleep-related gains (losses) during the initial sleep period determined from the difference in TT between the 6-hr and 24-hr test (sleep1), as well as a second sleep period assessed between the 24-h and 72-h tests (sleep2)^15,16^. The time-dependent offline gain (loss) was assessed using a 4 (Condition: RP-S, IP-S, L-PMd, R-PMd) between-subject ANOVA on the DS. Offline gains (losses) following overnight sleep were assessed using a (Condition: RP-S, IP-S, L-PMd, R-PMd) × 2 (Test: sleep1, sleep2) mixed-model ANOVA with repeated measures on the last factor.

### Reporting Summary

Further information on research design is available in the Nature Research Reporting Summary linked to this article.

## Supplementary information

Reporting Summary

## Data Availability

The datasets are available upon request by contacting the corresponding author, Taewon Kim (kimtay85@gmail.com).
