# Supplementary material for: Improving online and offline gain from repetitive practice using anodal tDCS at dorsal premotor cortex
Source: NPJ Sci Learn. 2021 Oct 22;6:31. doi: 10.1038/s41539-021-00109-4 (PMC8536655; doi:10.1038/s41539-021-00109-4)
Supplement: Supplementary file 1 — Reporting Summary [file 41539_2021_109_MOESM1_ESM.pdf]

nature research

Corresponding author(s): Taewon Kim

Last updated by author(s): June 25, 2021

Reporting Summary

Nature Research wishes to improve the reproducibility of the work that we publish. This form provides structure for consistency and transparency in reporting. For further information on Nature Research policies, see our Editorial Policies and the Editorial Policy Checklist.

Statistics

For all statistical analyses, confirm that the following items are present in the figure legend, table legend, main text, or Methods section.

n/a	Confirmed	
☐	☒	The exact sample size (n) for each experimental group/condition, given as a discrete number and unit of measurement
☐	☒	A statement on whether measurements were taken from distinct samples or whether the same sample was measured repeatedly
☐	☒	The statistical test(s) used AND whether they are one- or two-sided<br>*Only common tests should be described solely by name; describe more complex techniques in the Methods section.*
☒	☐	A description of all covariates tested
☒	☐	A description of any assumptions or corrections, such as tests of normality and adjustment for multiple comparisons
☐	☒	A full description of the statistical parameters including central tendency (e.g. means) or other basic estimates (e.g. regression coefficient) AND variation (e.g. standard deviation) or associated estimates of uncertainty (e.g. confidence intervals)
☐	☒	For null hypothesis testing, the test statistic (e.g. F, t, r) with confidence intervals, effect sizes, degrees of freedom and P value noted<br>*Give P values as exact values whenever suitable.*
☒	☐	For Bayesian analysis, information on the choice of priors and Markov chain Monte Carlo settings
☒	☐	For hierarchical and complex designs, identification of the appropriate level for tests and full reporting of outcomes
☐	☒	Estimates of effect sizes (e.g. Cohen's d, Pearson's r), indicating how they were calculated

Our web collection on statistics for biologists contains articles on many of the points above.

Software and code

Policy information about availability of computer code

Data collection	Date was collected using E-Prime 2.0 (Psychology Software Tools, Inc., Sharpsburg, PA, USA).
Data analysis	All statistical analyses were performed with STATA V15.1(Stata Corp LP, College Station, TX).

For manuscripts utilizing custom algorithms or software that are central to the research but not yet described in published literature, software must be made available to editors and reviewers. We strongly encourage code deposition in a community repository (e.g. GitHub). See the Nature Research guidelines for submitting code & software for further information.

Data

Policy information about availability of data

All manuscripts must include a data availability statement. This statement should provide the following information, where applicable:

- Accession codes, unique identifiers, or web links for publicly available datasets
- A list of figures that have associated raw data
- A description of any restrictions on data availability

The datasets are available upon request by contacting the corresponding author.

Field-specific reporting

Please select the one below that is the best fit for your research. If you are not sure, read the appropriate sections before making your selection.

☐ Life sciences ☒ Behavioural & social sciences ☐ Ecological, evolutionary & environmental sciences

For a reference copy of the document with all sections, see nature.com/documents/nr-reporting-summary-flat.pdf

Behavioural & social sciences study design

All studies must disclose on these points even when the disclosure is negative.

Study description	Behavioral experiment with separate experimental conditions.
Research sample	Participants were recruited from the Texas A&M University, (US). Participants were right-handed undergraduate (between 19-23 years old) students (N = 64, Males: 29, Females: 35).
Sampling strategy	Sample sizes were based on previous research with similar protocols, and assumed the power of 85% and type I error of 0.05, a total 64 subjects were sufficient in this study.
Data collection	Individuals had no prior experience with the experimental tasks and were unaware of the specific purpose of the study. All participants completed an informed consent approved by an IRB before any involvement in the experiment.
Timing	2019 - 2019
Data exclusions	All individuals that participated in this study had no history of epilepsy, any known neurological disorder, no psychiatric history, were medication free during the previous 14-days prior to participation, had not used alcohol within previous 24-h and were not pregnant.
Non-participation	No participants declined participation
Randomization	Based on the between subject experimental design in the present study, participants were randomly assigned to one of four (R-PMd, L-PMd, RP-S, IP-S) experimental conditions.

Reporting for specific materials, systems and methods

We require information from authors about some types of materials, experimental systems and methods used in many studies. Here, indicate whether each material, system or method listed is relevant to your study. If you are not sure if a list item applies to your research, read the appropriate section before selecting a response.

Materials & experimental systems

n/a	Involved in the study	
☒	☐	Antibodies
☒	☐	Eukaryotic cell lines
☒	☐	Palaeontology and archaeology
☒	☐	Animals and other organisms
☐	☒	Human research participants
☒	☐	Clinical data
☒	☐	Dual use research of concern

Methods

n/a	Involved in the study	
☒	☐	ChIP-seq
☒	☐	Flow cytometry
☒	☐	MRI-based neuroimaging

Human research participants

Policy information about studies involving human research participants

Population characteristics	See above
Recruitment	All participants were recruited from Texas A&M University community, and received either course credit for participation.

Ethics oversight	The studies involving human participants were reviewed and approved by the Institutional Review Board at the Texas A&M University. The participants provided their written informed consent to participate in this study.

Note that full information on the approval of the study protocol must also be provided in the manuscript.
